# Dietary Intake Estimated From a 24-Hour Recall Questionnaire in the Dene and Métis Communities of the Northwest Territories, Canada

**DOI:** 10.1016/j.cdnut.2023.100055

**Published:** 2023-02-21

**Authors:** Mylène Ratelle, Kelly Skinner, Maria Ramirez Prieto, Brian Douglas Laird

**Affiliations:** School of Public Health Sciences, University of Waterloo, Waterloo, Canada

**Keywords:** dietary intake, nutrient intake, nutrient deficiency, Indigenous peoples, First Nations, Dene, country foods, traditional diet, Northwest Territories

## Abstract

**Background:**

Food security and nutrient deficiencies are frequent issues for people living in northern remote regions of Canada.

**Objective:**

The objective of this study is to describe the nutrient intake of residents living in the Dene/Métis communities of the Dehcho and Sahtú regions of the Northwest Territories.

**Methods:**

A 24-h dietary recall survey was used to collect information from participants of a study completed in 9 communities during the winter seasons of January 2016 to March 2018. Intakes for food groups, vitamins, macroelements, and microelements were calculated. Nutrient intakes were compared with the available DRIs.

**Results:**

In total, there were 197 participants. On average, 37% of their energy was consumed from fat, and fruit/vegetable consumption was low (2.8 servings). Some vitamin levels (i.e., folate and vitamins A, B-6, C, and D) indicated a risk of nutritional deficiency for at least half of the participants. Of the nutrients examined, the nutrients least likely to meet the DRIs, according to the age/sex category of respondents were vitamin D (6%–20%), fiber (0%–11%), and calcium (4%–30%). Males tended to have a higher rate of nutrient adequacy above the DRIs. Importantly, 52% of the childbearing age female participants appeared deficient in folate, 48% deficient in zinc, 41% deficient in B12, and 22% deficient in iron, which might affect pregnancy and children’s development.

**Conclusions:**

A focus on supporting a higher intake of nutrient-dense foods would benefit the health of these communities. Nutrition and health promotion programs should be implemented to improve public health efforts in the region.

## Introduction

Indigenous people (First Nations, Métis, and Inuit) living in remote subarctic and Arctic regions within Canada have a unique diet owing to their geographic isolation and remoteness of their communities [1,2]. Subsequently, food insecurity is a frequent issue in the North. Food security, as defined by the Food and Agriculture Organization of the United Nations, is “a situation that exists when all people, at all times, have physical, social and economic access to sufficient, safe and nutritious food that meets their dietary needs and food preferences for an active and healthy life” [3]. Notably, the Canadian Community Health Survey in 2017/2018 estimated that household food insecurity rates reach 13%, 16%, and 49% in the Yukon, the Northwest Territories (NWT), and Nunavut, respectively [4].

Similarly, in the Sahtú region, preliminary work indicated that almost all households surveyed had experienced food insecurity at some point in the previous year [5]. In addition, dietary patterns and nutrient intakes in northern Indigenous populations often vary by seasons and food sources [1,6].

**FIGURE 1:**
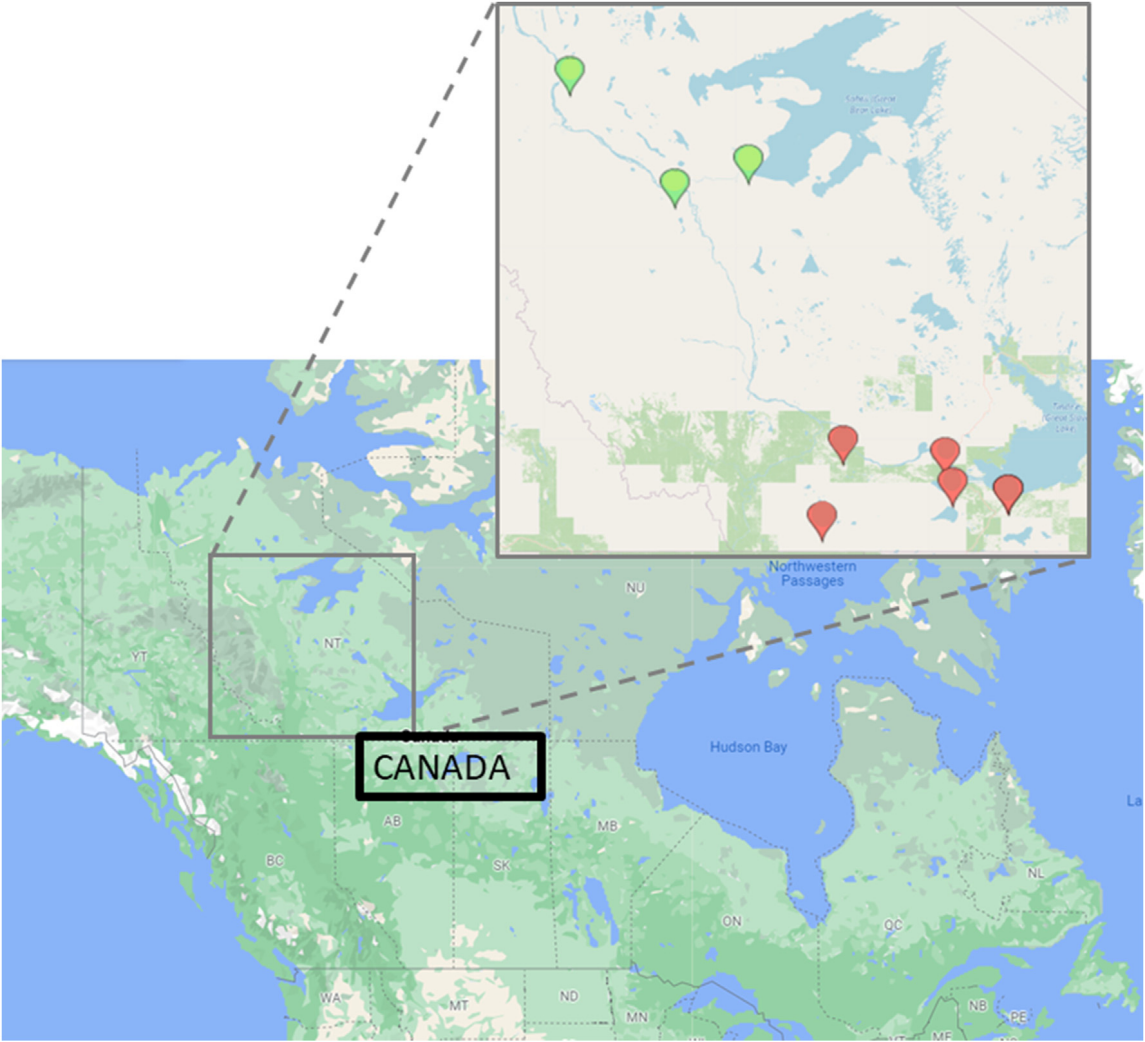
Participating communities in the 24-h recall survey, Northwest Territories, Canada, 2016–2018. Map made with Google mapping website. Green dots are the locations of the participating Sahtú communitie, and red dots are the locations of the participating Dehcho communities.

Food insecurity has been associated with lower levels of vitamins, such as vitamins A, B-1, B-2, B-6, B-12, and D [7,8]. In Canada, nutrient deficiencies identified among First Nations and Métis people of the NWT include vitamins A, C, D, and E, omega (ω)-3 and ω-6 fatty acids, calcium, folate, iron, magnesium, phosphorus, zinc, and dietary fiber [9]. Of these deficiencies, Indigenous populations living in the Arctic are at particularly high risk for vitamin D deficiency. For example, vitamin D deficiency is experienced by up to three-quarters of the Inuit population [10].

Country food (CF), which includes game meat, wild birds, fish, and foraged foods [also often referred to by the term traditional food (TF), consumed over several generations] [11], encompasses food ingredients and practices associated with a specific region or culture, and sourced locally. CFs are associated with a better nutritional status [12,13] and may benefit a food security status.

The Dene are also known as the Athapaskan peoples and live in a large geographical area of North America. Dehcho and Sahtú regions of the NWT, Canada, are part of the Land inhabited by Dene. These remote regions are located in a subarctic area, have a very low population density, and several of their communities are fly-in only. In addition, nutritional intakes and deficiency rates are currently not known among the Dene of the NWT. Accordingly, collaborative work was initiated in 2015 to identify the local environmental health priorities of Dene communities [14].

This article aimed to report nutritional data from a computer-based 24-h dietary recall survey collected in 9 Dene communities (see Figure 1). These findings are from the analysis of the data collected through a larger project to investigate the links between CF, nutrition, and contaminant intake, which are primary outcomes of the project.

Importantly, a 24-h dietary recall is a tool that has been widely used to assess the intake of food over a full day. Versions of our 24-h dietary recall survey have extensively been used for over 20 y in Canada [[15], [16], [17]], including with Indigenous populations [18,19].

## Methods

### Background: the Human Biomonitoring Project

First Nations communities in the Dehcho and Sahtú regions of the NWT participated in a biomonitoring project from 2016 to 2018 to study the links between nutrition, CFs, and contaminant exposure. The design and implementation of the larger biomonitoring project are provided in full detail elsewhere [14,20]. Six communities in the Dehcho and 3 in the Sahtú regions accepted to participate in the project. Participating First Nations (and communities) were as follows: Ts'ueh Nda (West Point), K'atl'odeeche First Nation (Hay River Reserve), Tthets'éhk'edélî (Jean Marie River), Deh Gáh Got'îê (Fort Providence), Sambaa K'e (Trout Lake), Ka'a'gee Tu (Kakisa), K'asho Got'ine (Fort Good Hope), Délınę (Deline, formerly Fort Franklin), and Tulít'a (Tulit’a, formerly Fort Norman).

Research agreements describing the scope of work, data management ownership, expected benefits, and outcomes were created with each participating community. Samples and survey data were collected by the local research coordinators and members of the research team who traveled to the participating communities. Recruitment to participate in the larger study was through posters, word of mouth, and local radio interviews, and for larger communities of more than 100 residents, randomly selected households were called by telephone to invite all residents in the household to participate. Community members were invited to come to a biomonitoring “clinic” where the samples and survey data were collected. Community members aged 6 y and older were eligible to participate in the project. In addition, to follow a more inclusive approach, walk-in participants were welcomed at the sampling clinic.

Participants provided informed consent and authorized the use of their data in published research. Compensation for participation was given in the form of a gift card to the local general store. Further details on recruitment, consent, and data management can be found elsewhere [14,20].

Participants who chose to partake in the study were asked to sign a consent form and provide basic demographic (e.g., sex and age) and personal characteristics (e.g., height, weight, and smoking status). Participants could choose 1, a few, or all 6 study components that involved the collection of blood, urine, and hair samples, 2 dietary surveys (a 24-h dietary recall and a food frequency questionnaire), and a Health Messages Survey examining the awareness and risk perception of advisories and preferred sources of health and contaminant information. In particular, the 24-h dietary recall was the focus of this article.

### The 24-h dietary recall survey

The design of the 24-h recall is provided in full detail elsewhere [13]. Through a web-based 24-h dietary recall survey, participants provided details on what they had eaten over the previous 24 h [16]. When participants began the survey, they selected foods by searching from a list of more than 900 food and beverage options or by choosing a food category. Prompts were built in to ask participants about several common forgotten items (e.g., sugar in coffee). Photographs of various portion sizes assisted in the appropriate estimation of foods consumed. Energy and nutrient content were integrated into the survey using the Canadian Nutrient File Database [21] and analyzed with the built-in assistance of the Elizabeth Stewart Hands and Associates Food Processor Nutrition Analysis Software (version 7.9; Esha Research). The food group serving were analyzed online according to the Canadian Nutrient File descriptions [21]. Although the participant was invited to complete the survey alone after a brief introduction, the members of the research team were available to help participants if needed. On average, participants took less than 20 minutes to complete the survey.

### Analysis

Results were calculated from the dietary intake reported from the previous day. This article collected aggregated data for the 9 communities and some disaggregated data for the Dehcho and Sahtú regions by sex and age. Age cutoffs were selected as follows: 6–17, 18–50, and ≥51 y. The age groups were chosen based on the following: *1*) the aim to have a sample size of >40 participants per group; *2*) physiologic considerations [young adults (18+) compared with older adults]; and *3*) aligning with age groups selected for the Canadian DRI estimate (cutoff of 51 y). In addition, age groups to assess deficiencies were selected based on a comparative study [22] and based on a value close to the median age. Descriptive statistics (i.e., arithmetic means, frequencies, and percentages) were calculated for energy intake, food groups, macronutrients, and micronutrients (i.e., kilocalories, protein, carbohydrate, fiber, sugar, fats, vitamins A, B-1, B-2, B-3, B-6, B-12, C, and D, folate, calcium, iron, potassium, selenium, sodium, zinc, and ω-3). Data addressing food group consumption were examined separately. Food groups (classification and serving) were based on Canada’s Food Guide [23] and included grain products, vegetables and fruit, milk and alternatives, meat and alternatives. Data analysis was performed using Microsoft Office Excel. In addition, an exploratory Pearson correlation investigation was performed using Microsoft Office Excel to estimate the relationship between variables (e.g., age, sex, and region) and nutritional intake. The statistical significance (*P* value) was not assessed.

### Research and ethic licenses

Ethics approval was obtained from the University of Waterloo Research Ethics Committee (#20173, #20950), the Stanton Territorial Health Authority for Human Research (29 December 2015), the Aurora Research Institute (#15560, #15775, #15966, #15977, #16021), and Health Canada (REB 2016-0022). Community research agreements were established between the research team and each participating community in the study (see the Background: the Human Biomonitoring Project section).

## Results

### Participant characteristics

Among the 199 participants completing the 24-h dietary recall, 2 participants were removed from the data set and excluded from the data analysis because their reported energy intake was extreme and outside a normal range (i.e., 10 and 10,595 kcal). Therefore, 197 participants were included in this study. Participants ranged in age from 7 to 85 y, with a mean age of 39.9 y (Table 1). The mean age among participants from the Dehcho region was 39.1 and 41.5 y in the Sahtú and 11% of the participants were aged younger than 18 y. The participation of females (50.3%) was similar to that of males (49.7%). Demographics of participants by sex and age range were similar to the census data from these regions.

**TABLE 1:** Sex and age range of participants (n = 197)

		Males, *n* (%)					Females, *n* (%)				
Region	Age^1^	7–17	18–50	51+	Unknown^2^	Total	7–17	18–50	51+	Unknown^2^	Total
Sahtú	65 (34)	8 (13)	9 (13)	14 (21)	1 (1)	32 (48)	35 (10)	15 (22)	12 (18)	1 (1)	35 (52)
Dehcho	127 (66)	13 (10)	31 (24)	17 (16)	1 (1)	66 (50)	14 (11)	31 (24)	17 (13)	2 (2)	64 (50)
Total	192 (100)	21 (11)	40 (21)	35 (18)	2 (1)	98 (49)	21 (11)	46 (24)	29 (15)	3 (1)	99 (51)
Mean age (y)^1^		11.4	37.2	64.4		41.5	12.8	36.3	60.0		38.3

1 Total of 5 participants did not provide their age: 3 from the Dehcho (1 male and 2 females) and 2 from the Sahtú (1 male and 1 female).

2 The participants did not provide this information.

### Dietary estimates and age/sex determinants

The mean of energy consumed in the previous 24 h (*n* = 197) was 1965 kcal (range, 284–4891 kcal), with most energy values between 1500 and 2000 kcals and 37% of the energy being from fat (total fat).

The mean of Canada Food Guide servings for vegetables and fruit, grain products, milk and alternatives, and meat and alternatives consumed are reported in Table 2. Vegetables and fruit consumption tended to be up to 3 times lower than recommended by the Eating Well with Canada’s Food Guide for First Nations, Inuit, and Métis; a mean of 2.8 servings are consumed, but 7–10 servings are recommended for adults [23]. The consumption of grain products was higher than vegetables and fruit for all categories (mean: 2.8 and 4.9 servings, respectively). Other food groups, usually high in fats, were the most consumed food servings for all categories (8.1 servings) in this study. The mean intake of energy was higher within the older age group. As expected, males tended to consume, on average, 22% more energy than females.

**TABLE 2:** Estimated intake (mean) of energy and food groups categorized by region, sex, and age for all participants (*n* = 197)

	Energy (kcal)	Grain products (servings)	Vegetables and fruit (servings)	Milk and alternatives (servings)	Meat and alternatives (servings)	Other food groups (servings)
All	1965	4.9	2.8	1.2	3.2	8.1
Dehcho	1929	4.5	2.7	1.3	3.0	7.7
Sahtú	2036	5.5	3.0	1.1	3.5	8.8
Females	1772	4.4	2.8	1.1	2.9	7.0
Males	2161	5.3	2.9	1.4	3.4	9.2
6–17 y	1737	5.2	2.7	1.2	1.9	5.1
18–50 y	1982	4.6	2.8	1.3	3.2	8.2
51+ y	2155	5.1	3.1	1.2	4.0	9.9

According to the Canadian Food Guide (Health Canada, 2010//), fortified soy beverage is an alternative to milk, and peanut butter, beans, lentils, and tofu are alternatives to meat.

Mean intake (in grams) of protein and fiber also varied according to age and sex, with consistently higher consumption by males and by older age categories. However, older adults (51≥ y) consumed less carbohydrates and sugar. Those aged 51 y or older ate the least amount of sugar and carbohydrates and the most protein and fiber, indicating a potentially healthier diet. The results are found in Table 3. In line with the servings from other food groups representing 40% of the daily food servings, the energy from fat represented 38% of the total consumed energy. Fats and cholesterol intake were higher with age. Among the older adults, cholesterol intake was noted to be 20% greater than that by younger adults.

**TABLE 3:** Estimated intake (mean) of protein, carbohydrate, fiber, sugar, cholesterols and energy from fats categorized by region, sex and age for all participants (n = 197)

	Protein (g)	Carbohydrate (g)	Fiber (g)	Sugar (g)	Cholesterol (mg)	E^1^ from fat (%)
All	95	212	13	73	434	38
Dehcho	91	207	13	75	434	38
Sahtú	102	223	13	69	430	36
Females	86	195	12	68	382	37
Males	103	230	14	78	485	38
6–17 y	70	216	12	80	289	33
18–50 y	92	215	13	78	439	38
51+ y	117	214	14	64	526	39

1 Energy (in kilocalories).

The means of vitamins and minerals are reported in TABLE 4, TABLE 5. With the exception of vitamin C intake, male’s mean intake for vitamins and minerals was higher than female’s intake, with the main differences occurring in vitamin B-6 intake status (B-6: 17.9 and 1.4 mg, respectively).

**TABLE 4:** Estimated intake (mean) of vitamins categorized by region, sex, and age for all participants (n = 197)

	A-RAE (RAE)	A-RE (RE)	B1 (mg)	B2 (mg)	B3 (mg)	B3-NE (mg)	B6 (mg)	B12 (μg)	C (mg)	D (IU)	D (μg)	Folate (μg)	Folate_DFE (μg)	Folate NAT (μg)
All	505	315	1.5	2.2	22	39	9.6	7.2	79	186	4.6	102	313	153
Dehcho	488	342	1.4	2.2	20	37	13.7	7.0	73	187	4.6	97	295	148
Sahtú	538	261	1.6	2.2	25	43	1.7	7.6	89	184	4.5	112	347	168
Females	470	264	1.3	1.8	20	35	1.4	6.5	79	149	3.7	92	291	140
Males	542	366	1.7	2.7	24	42	17.9	7.9	78	223	5.5	113	335	168
6–17 y	380	267	1.4	1.7	19	31	1.3	4.2	80	123	3.0	137	286	115
18–50 y	509	310	1.4	2.3	22	38	1.5	6.8	71	194	4.8	94	312	160
51+ y	593	352	1.7	2.5	25	46	26.6	9.9	91	221	5.4	94	341	177

DFE, dietary folate equivalent; IU, international unit; NAT, natural; NE, niacin equivalent; RAE, retinol activity equivalent; RE, retinol equivalent.

**TABLE 5:** Estimated intake (mean) of minerals categorized by region, sex, and age for all participants (n = 197)

	Calcium (mg)	Iron (mg)	Potassium (mg)	Selenium (μg)	Sodium (mg)	Zinc (mg)	DHA+EPA (mg)
All	640	14	2474	116	2929	13	33
Dehcho	649	13	2367	114	2971	13	34
Sahtú	624	15	2681	121	2849	12	31
Females	555	13	2197	97	2710	11	31
Males	727	15	2754	136	3151	14	36
6–17 y	552	13	1850	94	2695	10	14
18–50 y	649	14	2456	119	3042	12	33
51+ y	708	16	2960	133	2990	15	51

DHA and EPA are ω-3 fatty acids.

This difference in vitamin B-6 intake was also identified between age groups, with a much higher intake by older adults than that by younger adults (1.3–1.5 and 26.6 mg, respectively). Investigating the type of foods associated with higher levels of vitamin B-6 for participants, fortified cereals (e.g., Bran flakes) were observed to be a key element for higher vitamin B-6 level, and mostly consumed by males in the Dehcho. In addition, children showed higher intakes of folate compared with adults (137 and 84 μg, respectively).

Moreover, small differences between the Sahtú and Dehcho regions were observed. Sahtú participants tended to have higher intakes of protein, carbohydrates, and fiber, but Dehcho participants tended to have higher intakes of sugar and energy from total fats (%). Although there are some differences in intake in vitamins, the main one was lower vitamin B-6 intake higher in the Sahtú than that in the Dehcho (1.7 and 13.7 μg, respectively). In addition, a higher intake of vitamin C, folate, iron, potassium, and selenium was reported in the Sahtú, whereas a higher level of calcium was reported in the Dehcho. Notably, the higher level of calcium levels in the Dehcho than that in the Sahtú might be explained by Dehcho participants reporting a higher mean consumption of milk and alternatives than the Sahtú participants.

Furthermore, an exploratory investigation was performed to estimate the relationship between age and nutritional intake. However, a low positive Pearson correlation was observed between age and some of the nutritional elements, such as energy intake (*r* = 0.24) and protein intake (*r* = 0.37). Several vitamins were also associated with age. For instance, vitamin B-12 intake had the highest correlation (*r* = 0.34).

### Dietary deficiency estimates

Estimated nutrient intakes of adult participants on a single day are reported in TABLE 6, TABLE 7. The calculated mean intake of vitamins and minerals was compared with the DRIs [24] to assess the proportion of people at risk for nutritional deficiencies. Cutoff points were selected based on similar work performed in the region >20 y ago [12] and with a target sample size of at least 20 participants in each age category.

**TABLE 6:** Estimated intake (mean) of nutrients for adult participants (19 y and older) and estimated average requirements percentage (n = 153)

Age (y)	Median	EAR	% ≥EAR	Median	EAR	% ≥EAR
	Protein (g)^1^			Niacin/vitamin B-3 (mg)		
F ≤ 40	75	38	78	20	11	67
F > 40	90	38	86	19	11	74
M ≤ 40	71	46	75	17	12	60
M > 40	127	46	96	27	12	89
	Iron (mg)			Vitamin B-6 (mg)		
F ≤ 40	11.6	8.1	78	1.1	1.1	48
F > 40	11.2	5.0	90	1.2	1.3	37
M ≤ 40	10.3	6.0	90	1.2	1.1	55
M > 40	15.0	6.0	98	1.9	1.4	70
	Selenium (μg)			Vitamin B-12 (μg)		
F ≤ 40	73	45	84	3.8	2.0	59
F > 40	89	45	77	5.0	2.0	86
M ≤ 40	94	45	89	5.1	2.0	90
M > 40	161	45	94	6.9	2.0	96
	Zinc (mg)			Vitamin C (mg)		
F ≤ 40	3.7	6.8	52	46	60	44
F > 40	9.8	6.8	71	35	60	45
M ≤ 40	9.5	9.4	55	28	75	20
M > 40	14.0	9.4	83	48	75	37
	Vitamin A (μg)/RAE			Vitamin D (μg)		
F ≤ 40	427	500	48	2.5	10.0	15
F > 40	413	500	45	2.6	10.0	6
M ≤ 40	283	625	15	2.8	10.0	15
M > 40	653	625	52	5.2	10.0	20
	Thiamine/vitamin B-1 (mg)			Folate (μg DFE)		
F ≤ 40	1.3	0.9	78	315	320	48
F > 40	1.1	0.9	65	308	320	39
M ≤ 40	1.2	1.0	60	214	320	35
M > 40	1.6	1.0	85	325	320	50
	Riboflavin/vitamin B-2 (mg)					
F ≤ 40	1.8	0.9	82			
F > 40	1.6	0.9	88			
M ≤ 40	1.4	1.0	80			
M > 40	2.7	1.0	96			

Assumed DRI [24] values were because of age cutoff points. According to the cutoff variation, the 19–30 provided the DRI for ≤40 and the 51–70 provided the DRI for >40. F ≤ 40 y: *n* = 27; F > 40 y: *n* = 49, M ≤ 40 y: *n* = 20, M > 40 y: *n* = 54.

DFI, dietary folate equivalents; EAR, estimated average requirements; F, female; M, male; RAE, retinol activity equivalent.

1 Protein: based on 0.66 g/kg/d. Calculated for 57 kg for females and for 70 kg for males [22].

**TABLE 7:** Estimated nutrient intakes of adult participants (19 y and older) and AI percentage (n = 153)

Age1 (y)	Median	AI	% ≥AI	Median	AI	% ≥AI
	Carbohydrate (g)			Sodium (mg)		
F ≤ 40	191	130	82	2397	1500	78
F > 40	168	130	71	2303	1500	74
M ≤ 40	201	130	75	2150	1500	70
M > 40	146	130	91	3415	1500	85
	Fiber (g)			Potassium (mg)		
F ≤ 40	10	25	11	2169.0	2600	33
F > 40	9	21	10	1996.0	2600	33
M ≤ 40	11	38	0	1841.1	3400	10
M > 40	15	30	6	3293.3	3400	46
	Median	RDA	% ≥RDA			
	Calcium (mg)					
F ≤ 40	442	1000	11			
F > 40	537	1200	4			
M ≤ 40	472	1000	5			
M > 40	854	1000	30			

Assumed DRI [24] values were because of age cutoff points. According to the cutoff variation, the 19–30 provided the DRI for ≤ 40 and the 51–70 provided the DRI for >40. F ≤ 40: *n* = 27; F > 40: *n* = 49, M ≤ 40: *n* = 20, M > 40: *n* = 54.

The proportion of participants meeting the DRI for nutrients on the day of the 24-h recall was increased (at least 75% over the DRI for all the age/sex categories) for protein, iron, selenium, and vitamin B-2. The proportion of participants meeting the DRI was moderate (50%–75% over the DRI for all age/sex categories) for zinc and vitamins B-1, B-3, and B-12. However, some nutrient levels (i.e., folate and vitamins A, B-6, C, and D) indicated a risk of nutritional deficiency for at least half of the participants.

Older males (≥40 y) tended to have a higher rate of nutrients above the DRI. The nutrient least likely to meet the DRI was vitamin D, with the DRI for vitamin D met by only 6 to 20% of the participants. In particular, females aged ≥40 y had the lowest proportion meeting or exceeding DRI, followed by females younger than 40 y, males younger than 40 y, and males aged 40 y and older.

Similarly, macroelement levels were also low (e.g., fiber, calcium, and potassium). The group with the most risk of levels below the AI were males younger than 40 y. In particular, no male participant younger than 40 y met the AI for fiber.

Although the general nutrient weight intake (in milligrams) was less for females and younger adults, the young female category (aged 18–40 y) were less likely to have deficiencies in protein, vitamins A, B-1, B-3, B-6, and C, folate, and selenium than males of similar age. However, young women of childbearing age were still more likely to be deficient in iron and B12 than men.

### Contribution of CF to vitamin intake

The mean intake of vitamins and micronutrients according to participants who consumed CF is reported in Table 8. On the day CFs were consumed, the intakes of vitamins were systematically higher, including almost the double intake of vitamin D. However, the mean age for participants consuming CFs was 48 y and for those participants not consuming CF on the day of the survey was 37 y. As observed in TABLE 2, TABLE 3, TABLE 4, TABLE 5, TABLE 6, TABLE 7, the oldest age group was associated with higher intake of several nutrients, which might partially explain those results.

**TABLE 8:** Estimated intake (mean) according to the consumption of CFs (*n* = 197)

Vitamins and micronutrients	With CF	Without CF	CF/no CF (%)
*n*	62	135	
Calcium (mg)	764	584	131
DHA+EPA (mg)	0.64	0.20	324
Folate (μg)	100	103	97
Folate DFE (μg)	347	297	117
Folate NAT (μg)	175	144	121
Iron (mg)	19	12	159
Potassium (mg)	3264	2111	155
Selenium (μg)	128	110	116
Vitamin A (RAE)	594	465	128
Vitamin A (RE)	361	293	123
Vitamin B-1 (mg)	1.6	1.4	112
Vitamin B-2 (mg)	2.8	1.9	142
Vitamin B-3 (mg)	26	20	132
Vitamin B-3-NE (mg)	50	33	151
Vitamin B-6 (mg)	1.9	1.4	134
Vitamin B-12 (μg)	13.6	4.2	321
Vitamin C (mg)	98	70	141
Vitamin D (IU)	276	144	191
Vitamin D (μg)	6.8	3.5	193
Zinc (mg)	18.2	9.9	184

CF, country food; DFE, dietary folate equivalents; IU, international units; NAT, natural; NE, niacin equivalents; RAE, retinol activity equivalent; RE, retinol equivalent.

## Discussion

### Limits of the data interpretation

This research is based on a cross-sectional study. The data collection only includes data from a single 24-h recall and the interpretation is limited to the single day of the data collection. A repetition of the 24-h recall would provide strength in the data analysis among the participants. In addition, repeated 24-h recalls are needed to account for the intra-individual variation. The aim of this work was not to look at the individual outcome but at the community outcome, and hence, these findings are still very valuable for populations where any nutrition data are minimal. In addition, the small sample size prevents a generalization to the Dehcho and Sahtú population.

Limitations of the 24-h dietary recall include recall error, underreporting of foods, and inaccurate estimation of portion sizes [25]. The design of the 24-h dietary recall survey used in this study and its limits were extensively detailed elsewhere [13]. Importantly, the survey has several techniques to minimize the weaknesses of this form of assessment [15,16]. For example, the anonymity and confidentiality of the survey may promote more honesty in reporting sensitive data [16].

It is worth noting that 11% of these participants reported consuming vitamins, fiber, or other supplements but did not provide details on the brand, type, or concentration of those supplements. These additional vitamin intake were not integrated into the current survey tool. Hence, the deficiencies identified in this 24-h dietary recall survey could be overestimated. However, when comparing levels between participants reporting or not consuming vitamins and supplements, it seems that participants consuming supplements have higher levels of fiber (1% higher), vitamin A (IU) (237%), vitamin A (retinol activity equivalent) (4%), vitamin D (5%), and calcium (6%), but lower levels in all other dietary nutrients. It is unclear whether these participants used supplements to cope with poor nutrition or, on the contrary, to have healthier habits.

### Comparison with other studies

Recent work was performed by Batal et al. [26,27] in First Nations communities across Canada living south of the 60th parallel with the aim to assess dietary intakes using a 24-h recall survey. Similar to this study, they reported that men ate 23% more energy than women (18–50 y) and had higher nutrient weight intakes. The percentage of energy as fat was in the mid-thirties for both men and women, and the mean serving of vegetables and fruit was 2.8, being the same value as observed in this study. Calcium, vitamin A, and D were also the nutrients with the highest deficiency rates. Although those communities are located south of the communities included in this manuscript and are exposed to a warmer climate and a longer summer, deficiency rates of vitamin D were still between 95 % and 100% [26,27].

Other population dietary intakes located south of the NWT have also been assessed using a 24-h dietary recall. In the US adult population, for calcium, 31%–65% of men and 38%–44% of women met AI recommendations based on age. For vitamin D, 24%–59% of men and 22%–56% of women met AI recommendations [28]. The aforementioned statistics paint a healthier nutritional population portrait than this study population. In Canada, north of the United States, the Canadian Community Health Survey (2004) also used a 24-h dietary recall to assess nutrient intakes from food [29,30]. The inadequacy of the estimated average requirement in Canadian adults applied to almost half of the adults for vitamin A and calcium intake. For vitamin D, >90% of adults were likely to have intake below the estimated average requirement. In 2004, for Indigenous people (19–50 y) living off-reserve in the provinces (excluding the Territories), the inadequacy rates for vitamin D for men and women were 96% and 93%, respectively, and the inadequacy rates for vitamin A were 71% and 58%, respectively [30]. Similar levels of nutritional deficiency prevalence are still found in northern Indigenous communities, as indicated in this study. However, calcium inadequacy rates are currently much higher than those for Indigenous people living in the southern provinces.

Erber et al. [31] assessed the dietary adequacy in a remote Inuvialuit population of the Northwest Territories. Most adults did not meet the recommended intake of vitamins A, B-6, and E, fiber, calcium, and folate. As per our study, vitamin D intake was below the recommendation for most women.

Although our study does not report deficiencies in children owing to the small sample size, other regions in Northern Canada reported a high prevalence of vitamin D deficiency in children. For instance, 97% of Inuit preschoolers were found to be vitamin D deficient when sampled in the winter [32].

Similarly, Kuhnlein et al. [22] used a similar 24-h dietary recall approach in the 1990s to assess nutrient intake and CF consumption of Dene communities of the NWT (*n* = 1007) during the 2 seasons. Similar to this study, the authors reported their data by women aged 40 y and younger and older than 40 y and men aged 40 y and younger and older than 40 y. Although the data cannot be directly compared, we estimated that the dietary requirement rates were similar to those in the 1990s in the same region [13]. It is worth noting that using a single survey on a winter day might overestimate the issue of nutrition deficiencies because summer is usually associated with increased access to TF.

In addition, a study was undertaken about 10 y ago to determine the level of dietary vitamin D in a Dene community (*n* = 46) using a food frequency questionnaire [33]. Based on the 2011 RDA values, they reported that 11% of participants met the RDA value for vitamin D in the winter. The observed low prevalence of vitamin D intake was similar to what was observed in this study.

In another study within the Sahtú region, a subsample of blood plasma samples was analyzed for nutrition biomarkers [5]. Vitamin D (25-hydroxyvitamin D3) and vitamin B-9/folate (5-methyl THF) were the markers associated with the highest deficiency rates for participants. Similarly, at the time of the sample collection, more than half of the participants seemed to be severely deficient in 25-hydroxyvitamin D3 (below 10 ng/mL). This study showed similar results to the wide deficiency occurrence of vitamin D in the Dene population.

### Public health perspectives

From a public health perspective, nutritional deficiencies are associated with multiple health consequences, such as metabolic syndrome [34], immune disorders [35], mental health issues [36], reproductive health [37], and more.

The findings indicate the importance of improving intake of vitamin D, fiber, calcium, and folate. To address these challenges in nutrient deficiency, several options are possible, such as food fortification, shelf tag interventions, nutrition education, or other health promotion efforts. A mix of technology-based and community-based nutrition education campaigns is worthy of interest (low cost and easily managed locally) for the Dehcho and Sahtú regions. In other populations, online interventions aimed at increasing knowledge of vitamin D resulted in significant increased dietary intake [38]. Nutritional education in person and by text messages improved the intake of folic acid supplements [39]. However, this type of intervention tends to focus on a single nutrient. A lower consumption of fat and sugar would also benefit the health of these communities.

It was reported that vitamin D supplements were an important contributor, potentially the main one, to achieve a minimal biological target for vitamin D for older people living at high latitudes [40]. It was shown that vitamin D food fortification increased 25(OH)D concentrations in adults, and hence, food-based strategies could prevent not only vitamin D deficiency [41] but also other vitamin deficiencies.

Moreover, TFs and CFs can improve the intake of important nutrients, play a role in combating food security, and improve diet quality. In remote Canadian regions where food security might be a challenge, a traditional diet was identified as an important determinant contributing to nutrient levels [[42], [43], [44], [45]]. Kenny et al. [46] reported that CFs for 3 northern Indigenous groups within Canada were a principal source of many micronutrients (18%–82%), such as iron, niacin, and vitamins D, B-6, and B-12. Previous work in this specific Dene population also indicated 47% higher protein intake and 7% less fat contribution to energy intake on the days where CFs were consumed [13]. Several studies indicate the preference of northern Indigenous people to consume more CFs but being unable to do so owing to several barriers [5,18,47,48]. Financial factors, equipment, industrial activities, regulations, climate change, contamination perceptions, and lack of knowledge were some of the barriers limiting the access to TFs/CFs [5,47,49].

Importantly, with !52% of women of childbearing age appearing to be deficient in folate, 48% deficient in zinc, 41% deficient in B12, 22% deficient in iron, the barriers to CF might affect children’s development and have potential consequences in maternal health and birth defects. All these micronutrient deficiencies are known to be associated with developmental issues, preterm birth, and low birth weight [50]. To improve the nutrition of expecting mothers, northern initiatives have been implemented over the years. For example, the Nunavik Regional Board of Health and Social Services assessed the Arctic Char Distribution Project for pregnant women [51].

A wide range of public health efforts could be initiated to improve the health of the population. Slater et al. [33] reported that the use of a 24-h dietary recall survey in a Dene population showed that supplements, milk, and local fish were positively associated with adequate vitamin D intake. In fact, milk and local fish were the major dietary sources of vitamin D. Batal et al. [26] also identified the biggest contributors to nutrient intake in Indigenous communities within Canada to as fish for vitamin D, milk for calcium, and vegetables for vitamin A.

Similarly, the promotion of TF/CF consumption and facilitation to access such food would be beneficial to food security and nutrition. Such initiatives are already happening locally, based on projects funded by the federal government such as Nutrition North Canada, or funding opportunities from the Climate Change and Health Adaptation Program and others. For example, a 5-y Indigenous food harvest program is ongoing in the Sahtú region, which also integrates a CF education communication strategy. At the territorial level, health programs such as the Food and Nutrition program [52], provide online fact sheets, guides, and resources promoting CFs for healthy living. For example, they recommend pregnant women to “eat cooked fish at least once a week” and have culturally relevant advice for each CF type such as “fish heads and bones are excellent sources of calcium” and “fish eggs are fair sources of iron.” Although these key messages are excellent, the communication efforts could be improved to better reach the targeted population.

TF/CF access has been associated with improved food security [18,53]. However, there is a lack of evidence-based nutrition programs for Indigenous or remote northern adult populations with the aim to improve nutritional status. Granted, there are several challenges in developing an evidence base for health promotion and food security programs [54], which have resulted in limited available knowledge on proven interventions for food security and nutrition status.

For instance, an intervention in the NWT provides evidence of an improved diet for Indigenous people [[55], [56], [57]]. A health promotion program integrating different strategies related to the promotion of healthier food preparation methods, and the communication of benefits related to CFs and healthier store bought options, resulted in a healthier diet and higher vitamins A and D intake [[55], [56], [57]].

Furthermore, a study on food pricing policies in remote Aboriginal and Torres Strait Islander community stores in Australia identified financial subsidies as the most common intervention and indicated that decision-making to determine potential interventions lacked the integration of research-informed evidence [58]. Durao et al. [59] identified cash transfers as evidence to improve access to food in low- and middle-income countries, but nutrition status was not assessed. Although agroforestry diversity was associated with nutritional benefits in Indigenous women of Jharkhand, India [60] and has the potential to contribute to land stewardship and food sovereignty efforts [61], agroforestry is not widely developed in the North and has obvious challenges (e.g., cold climate, slow plant growth, and lack of equipment). Nevertheless, food diversity and access to fresh food may affect the nutritional status of northerners.

Store-based interventions, financial strategies, nutrition education, and promotional interventions also showed great potential in other remote Indigenous communities of Aboriginal and Torres Strait Islander Australians [62]. Moreover, community-based approaches were beneficial to several nutrition interventions worldwide [[63], [64], [65]].

Finally, older men are the least likely demographic to experience nutritional deficiencies in comparison with the other groups. This situation could be associated with gender-based activities of going on the land to harvest, which is historically associated with food security, and TF/CF access, which may influence healthy nutrient intake, food preferences, and dietary behavior. Further work is required to identify the causes of age and gender consumption differences. With that being said, oral teachings and passing on Indigenous Knowledge to younger generations, as mentioned in the Dene Laws, are important for the participating communities and may influence TF/CF consumption, food preferences, and dietary choices. Hence, participation of older men and elderly in public health efforts to promote healthy nutrition in Indigenous communities might be beneficial.

### Conclusion

In the participating Dene population, vitamin D, fiber, and calcium intake were low, resulting in deficiencies for the majority of the participants. Poor nutritional status might be because of several complex and intersecting challenges experienced by northern Indigenous communities, such as the historical context of colonialism, remote food insecurity, and social and environmental inequities. Furthermore, with challenges such as climate change and COVID-19 pandemic effects [66], the issue of food security is increasing in Northern communities. Without appropriate nutritional programs, adequate nutrition status, which has been stagnant for 15 y, may decrease over time. Nutrition education, financial interventions, and store-food pricing policies should be put in place to facilitate access to market food, and culturally adequate initiatives, such as community harvest programs, should be put in place to facilitate better access to TFs/CFs.

## Data Availability

Restrictions apply to the availability of these data. Data were obtained from the University of Waterloo research team and are potentially available from the corresponding author with the permission of each of the participating First Nation communities, under the OCAP principles of First Nations for the Ownership, Control, Access, and Possession of their knowledge, data, and information [67].
